# Diagnosis and Management of *Borrelia turicatae* Infection in Febrile Soldier, Texas, USA

**DOI:** 10.3201/eid2305.162069

**Published:** 2017-05

**Authors:** Anna M. Christensen, Elizabeth Pietralczyk, Job E. Lopez, Christopher Brooks, Martin E. Schriefer, Edward Wozniak, Benjamin Stermole

**Affiliations:** Eglin Air Force Base, Valparaiso, Florida, USA (A.M. Christensen, E. Pietralczyk, C. Brooks, B. Stermole);; Baylor College of Medicine and Texas Children’s Hospital, Houston, Texas, USA (J.E. Lopez);; Centers for Disease Control and Prevention, Fort Collins, Colorado, USA (M.E. Schriefer);; Texas State Guard, San Antonio, Texas, USA (E. Wozniak)

**Keywords:** febrile illness, borreliosis, *Borrelia turicatae*, tick-borne diseases, tick-borne relapsing fever, *Ornithodoros turicata*, vector-borne infections, zoonoses, bacteria, Texas, United States

## Abstract

In August 2015, a soldier returned from field exercises in Texas, USA, with nonspecific febrile illness. Culture and sequencing of spirochetes from peripheral blood diagnosed *Borrelia turicatae* infection. The patient recovered after receiving doxycycline. No illness occurred in asymptomatic soldiers potentially exposed to the vector tick and prophylactically given treatment.

Tickborne relapsing fever (TBRF) was first reported in the United States >100 years ago but is often difficult to identify, given its rarity and variety of clinical presentations (*1*,*2*). We describe a case of culture-confirmed TBRF caused by *Borrelia turicatae* acquired by an Army soldier during a military training exercise in Texas, USA.

The patient was a 31-year-old white man with no relevant medical history. In August 2015, he sought care at the Eglin Air Force Base Hospital (Valparaiso, Florida, USA) with a 5-day history of fever (102°F), chills, and myalgias. He reported headache and nausea but no vomiting or diarrhea. He denied localized joint pain, redness, or swelling but had discomfort in both popliteal fossae.

The patient had recently returned to Florida after a 30-day Army exercise under austere conditions in western Texas (Technical Appendix Figure 1). Potential exposures included sleeping nude in a sleeping bag on the floor of an abandoned barn that had been cleared of infestation with rabbits, rodents, birds, and bats; having eaten boar that had been slaughtered, dressed, and cooked over an open flame; and consuming water procured from a well, bottled sources, and at times through a LifeStraw (http://lifestraw.com/). Approximately 1 week before admission, he had noted multiple skin lesions, including scattered, presumed insect bites along his left leg and a small lesion at his urethral meatus. He denied any history of genital lesions and had not seen any biting insects. After 6 days, the lesions spontaneously resolved.

In a Texas emergency department, the initial diagnosis was viral syndrome, and a rapid influenza test result was negative. The fever persisted despite administration of antipyretics. After 2 days, the patient returned to the hospital, where he received only symptomatic treatment. No tests were ordered. After another 2 days, he sought care from his unit physician. Laboratory tests showed marked thrombocytopenia with 16 × 10^9^ platelets/L (reference range 150–400 × 10^9^ platelets/L). Spirochetes were seen on peripheral blood smear (Technical Appendix Figure 2). He was referred for hospital admission. Physical examination findings were unremarkable: no splenomegaly, hepatomegaly, or rash. Blood cultures and serologic testing for rickettsiae, HIV, dengue virus, *Treponema pallidum,* and plasmodia produced negative results. Erythrocyte sedimentation rate (58 mm/h) and C-reactive protein level (>19 mg/L) were elevated. Electrolytes and transaminase levels were within reference ranges.

Serum samples collected at admission and 3 weeks later (≈5 and 26 days after illness onset, S1 and S2, respectively) were tested in parallel at the Centers for Disease Control and Prevention, National Center for Emerging and Zoonotic Infectious Diseases, Division of Vector-Borne Infectious Diseases (Fort Collins, CO, USA) by enzyme immunoassay and Western blot (IgM and IgG) for TBRF antibody reactivity. Seroconversion was demonstrated by rising enzyme immunoassay values (S1 = 0.79, S2 = 2.41; equivocal range 0.64–0.91) and separate IgM and IgG Western blots (Technical Appendix Figure 3). In addition, the samples demonstrated seroconversion (S1 = 0.91, S2 = 3.23; equivocal range 0.90 –1.09) against C6, an immunogenic antigen conserved among *Borrelia* spp. (*Borrelia burgdorferi* ELISA; Immunetics, Inc., Boston, MA, USA).

Spirochetes were successfully cultured, and genomic sequencing determined that *B. turicatae* was the causative agent (*3*). The patient improved rapidly with doxycycline, and platelet count normalized within 2 weeks. Ten asymptomatic soldiers with similar exposure were identified and prophylactically given doxycycline; 24 asymptomatic soldiers who had been in the area but not in the same barn as the patient were monitored closely. No additional illnesses were detected.

TBRF is a neglected and probably underdiagnosed disease. The vector, the *Ornithodoros turicata* tick, is endemic to Texas and Florida (*4*); but although published cases in Texas have been supported by serology for the TBRF group, exposure location, and tick collections (*4*,*5*), to the best of our knowledge, successful identification of *B. turicatae* in a human has not been reported. Previously, *B. turicatae* has been isolated only from ticks and canids in several areas of Texas (*4*–*6*).

The ecologic setting of the military exercises was predictable for high-risk exposure to the tick vector. TBRF attack rates >22% have been reported for group settings with sequelae severe enough to warrant hospitalization (*7*,*8*). Military training groups in Israel have declared certain caves off limits because of heavy tick presence (*9*) and have prophylactically administered doxycycline to those suspected to have been exposed (*7*). There has not been an association of Jarisch-Herxheimer reaction in asymptomatic patients receiving doxycycline (*7*), although this reaction is common during treatment of patients with active illness (*9*).

We identified several difficulties in epidemiologic awareness and diagnosis. There is overlap of bacterial, viral, and parasitic pathogens in location and nonspecific symptom presentations. The *O. turicata* tick bite is rarely noticed or reported because the vectors are rapid nocturnal feeders, attachment is painless, and often no lesions or ticks are discovered (*10*). This case report with successful isolation and genetic characterization of *B. turicatae* from the soldier (*3*) confirms that this spirochete species is a zoonotic pathogen. The initial misdiagnosis further indicates the neglected nature of this disease, especially in the military population. 

Technical Appendix Case of *Borrelia turicatae* infection in febrile soldier: time line, histologic appearance, and immunoblots**.**
