# Supplementary material for: Diagnosis and Management of Borrelia turicatae Infection in Febrile Soldier, Texas, USA
Source: Emerg Infect Dis. 2017 May;23(5):883–4. doi: 10.3201/eid2305.162069 (PMC5403040; doi:10.3201/eid2305.162069)
Supplement: Technical Appendix — Case of Borrelia turicatae infection in febrile soldier: time line, histologic appearance, and immunoblots. [file 16-2069-Techapp-s1.pdf]

Article DOI: http://dx.doi.org/10.3201/eid2305.162069

Borrelia turicatae Infection in Febrile Soldier, Texas, USA

Technical Appendix

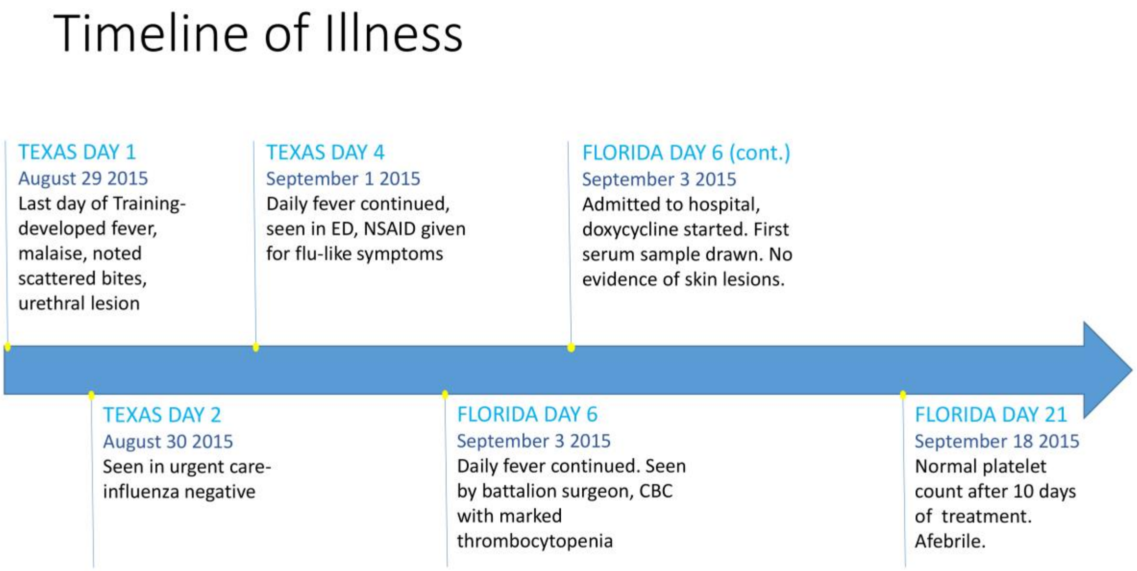

Technical Appendix Figure 1. Time line of *Borrelia turicatae* infection in man. ED, emergency department; NSAID, nonsteroidal anti-inflammatory drug; CBC, complete blood count.

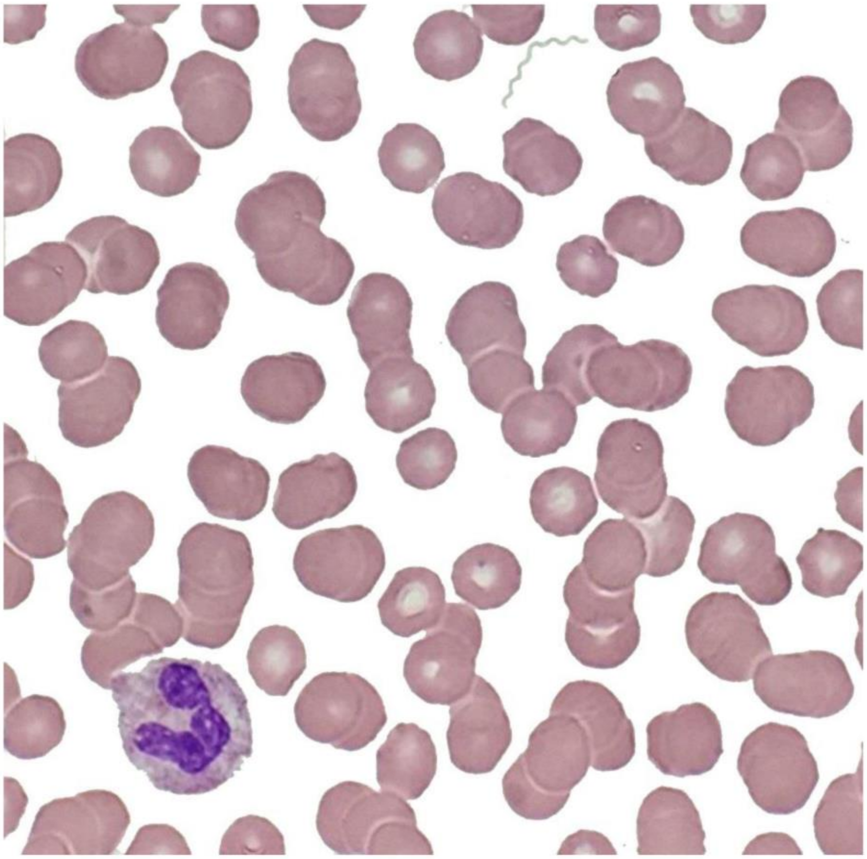

Technical Appendix Figure 2. *Borrelia turicatae* in a stained peripheral blood smear from febrile soldier, Texas, USA. Original magnification ×100, oil immersion.

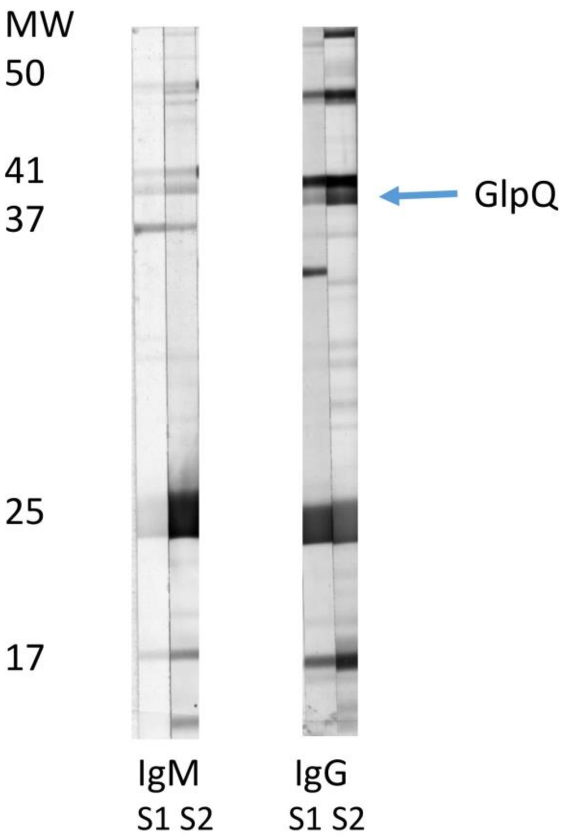

Technical Appendix Figure 3. Immunoblots demonstrating increasing tick-borne relapsing fever antibody reactivity to discrete antigens. GlpQ, glycerophosphodiester phosphodiesterase Q gene (serologic discriminator between relapsing fever and Lyme borreliosis); MW, molecular masses expressed in kilodaltons; S1, first serum sample collected day 5 of illness; S2, second serum sample collected 3 weeks later.
